# Prospective Comparison of Nonnarcotic versus Narcotic Outpatient Oral Analgesic Use after Laparoscopic Appendectomy and Early Discharge

**DOI:** 10.1155/2014/509632

**Published:** 2014-04-14

**Authors:** Fuad Alkhoury, Colin Knight, Steven Stylianos, Jeannette Zerpa, Raquel Pasaron, JoAnne Mora, Alexandra Aserlind, Leopoldo Malvezzi, Cathy Burnweit

**Affiliations:** ^1^Department of Pediatric Surgery, Joe DiMaggio Children's Hospital, 1150 North 35th Avenue, Suite 555, Hollywood, FL 33021, USA; ^2^Miami Children's Hospital, 3200 SW 60th Court, Suite 201, Miami, FL 33155, USA

## Abstract

*Purpose*. To compare narcotic versus nonnarcotic outpatient oral pain management after pediatric laparoscopic appendectomy. *Methods*. In a prospective study from July 1, 2010, to March 30, 2011, children undergoing laparoscopic appendectomy on a rapid discharge protocol were treated with either nonnarcotic or narcotic postoperative oral analgesia. Two surgeons in a four-person faculty group employed the nonnarcotic regimen, while the other two used narcotics. Days of medication use, time needed for return to normal activity, and satisfaction rate with the pain control method were collected. Student's *t*-test was used for statistical analysis. *Results*. A total of 207 consecutive children underwent appendectomy for acute, nonperforated appendicitis or planned interval appendectomy. The age and time to discharge were equivalent between the nonnarcotic (*n* = 104) and narcotic (*n* = 103) groups. Both had an equivalent number of medication days and similar times of return to normal activity. Ninety-seven percent of the parents of children in the nonnarcotic group stated that the pain was controlled by the prescribed medication, compared to 90 percent in the narcotic group (*P* = 0.049). *Conclusion*. This study indicates that after non-complicated pediatric laparoscopic appendectomy, nonnarcotic is equivalent to narcoticbased therapy for outpatient oral analgesia, with higher parental satisfaction.

## 1. Introduction

Previously, pain has been underestimated and undertreated in children, due to individual and social attitudes toward pain and the complexity of its assessment in children [1–3]. Over the past decade, the importance of pain control in the pediatric population has been more widely recognized, especially as outpatient procedures have been performed with increasing frequency; they currently constitute 60% to 70% of operative procedures conducted in North America [4]. Although no population-based data examining practice patterns of postoperative analgesia after ambulatory pediatric general surgical procedures exist, the current standard at many institutions is to provide a prescription for opioids, frequently oral acetaminophen plus codeine or oxycodone for analgesia after outpatient surgery. Nonsteroidal anti-inflammatory drugs (NSAIDs) have been widely studied and are proven to be effective analgesics for postoperative pain control [5, 6]. Unfortunately, no prospective trials have examined the combination of acetaminophen plus NSAIDs compared with acetaminophen plus narcotics in outpatient pediatric surgery procedures.

At the community-based children's hospital where this study took place, laparoscopic appendectomies were performed primarily as an outpatient procedure [7]. The objective of this study was to compare the efficacy of acetaminophen/ibuprofen (nonnarcotic) or acetaminophen plus codeine or oxycodone (narcotic) for management of pain in children undergoing laparoscopic appendectomy on a rapid discharge protocol.

## 2. Methods

After Institutional Review Board (IRB) approval, a prospective trial was carried out to evaluate postappendectomy pain control, comparing nonnarcotic to narcotic medications. Individual consents and assents for this study were waived as treatment options were presented as standard of care. Healthy children in the American Society of Anesthesia (ASA) risk classes 1 and 2 undergoing laparoscopic appendectomy at the community-based children's hospital using a rapid discharge protocol [7] between July 1, 2010 and March 30, 2011 were enrolled in the study. All subjects left the hospital within 24 hours of the surgery. Children having any additional procedure(s) at the time of the appendectomy, those with chronic medical issues (ASA class 3 or higher) or developmental delays, patients with allergies or contraindications to study medications, and those receiving chronic treatment with opioids or NSAIDs were excluded.

Children were divided based on clinical practice patterns, as two surgeons in a four-person faculty group employed the nonnarcotic regimen, while the other two routinely used narcotics. The nonnarcotic group received 15 mg per kg of acetaminophen every 4 hours as needed plus 10 mg per kg ibuprofen every 6 hours around the clock (ATC) for 48 hours and then every 6 hours as needed, while the narcotic group received 10 mg/kg acetaminophen plus 1 mg/kg codeine or oxycodone every four hours as needed for pain. During the course of the study parents were agreeable to the pain management treatment regime utilized by their child's surgeon and there were no instances of parents requesting alternate pain treatment methods.

At the time of the postoperative visit, parents were asked to document days of medication use and time needed for return to normal activity. Due to the high potential for subjectivity, pain scores were not utilized as an assessment parameter for this study. In addition, parents rated their satisfaction with the pain control method. Student's* t*-test and Fisher's exact test were used for statistical analysis.

## 3. Results

A total of 251 children, ranging in age from 2 to 19 years, underwent appendectomy over the nine-month study period. Two hundred fifteen patients had the presumed diagnosis of acute simple appendicitis and proceeded to surgery. The remaining 36 patients underwent interval appendectomy after medical management for perforated disease (15 of these had an exposed fecalith or residual abscess cavity and were admitted to postoperative intravenous antibiotics, while 21 underwent uncomplicated procedures and were eligible for expeditious discharge and inclusion in the pain control study).

Of the 215 that proceeded to surgery for laparoscopy, gangrene or perforation was noted in 29 patients (these children were admitted to antibiotics postoperatively and excluded from further study). The final study population consisted of a total of 207 children. Of the 207, the single-port, single-instrument transumbilical approach [8] was used in 198 patients (96%).

There was no difference in demographics and operative details when comparing children who received narcotics with those who did not (Table 1). The cohorts had equivalent number of medication days and similar times to normal activity. Ninety-seven percent of the parents of children in the nonnarcotic group stated that the pain was controlled by the prescribed medication, compared to 90 percent in the narcotic group (Table 2).

## 4. Discussion

In the past, effective pain alleviation in the pediatric field was often inadequate due to the misimpression of medical personnel and caregivers that analgesic drugs were harmful [9, 10] and that pain reception was muted in the young. Analgesia appropriate for the intensity of suffering should be provided both in the hospital setting and at home. Of late, more emphasis has been placed on the assessment and treatment of noxious stimuli in the practice of pediatrics, and the study of relief of pain in neonates, infants, and children has moved to the forefront [1, 2]. The goal of the present trial was to examine the efficacy of nonnarcotic versus narcotic regimens in postoperative pain control after laparoscopic appendectomy in children. Surprisingly, our work demonstrated that nonnarcotic medication was at least as effective as the “stronger,” opioid-based therapy (Table 2).

The discomfort associated with any abdominal surgery is sufficiently severe to merit postsurgical analgesia [11]. Because different procedures may induce incomparable levels of pain, we focused on a single operation in a prospectively observed setting—children on a rapid discharge pathway after appendectomy—to control both operative and postoperative variability, lending credibility to the comparison between the nonnarcotic and the narcotic therapy.

There was statistical difference in parents' satisfaction, who favor the nonnarcotic therapy in this study. Of note, in the nonopioid regimen, ibuprofen was recommended “around the clock” immediately following operation, not “as needed.” Although widely used by surgeons, as-needed administration of analgesia appears, in randomized trials, to be substandard when compared to regular dosing after ambulatory surgery [12].

In conclusion, nonnarcotic therapy when compared with narcotic therapy did not provide inferior analgesic, and it was associated with a higher parental satisfaction. This study failed to show any evidence to support the widespread use of opioids in children in the settings of early discharge after appendectomy. It would be reasonable to suspect that this regimen could be applied successfully in a range of outpatient procedures. The results of this trial suggest that a safe, effective, and inexpensive strategy for outpatient analgesia is a combination of acetaminophen and ibuprofen, a treatment option that avoids the possible complications of opioid use.

## Figures and Tables

**Table 1:** Demographics and operative details.

	Nonnarcotic (*n* = 104)	Narcotic (*n* = 103)	*P* value
Age years	11.2±	11.6±	0.94
Acute appendectomy	*n* = 94	*n* = 92	
Interval appendectomy	*n* = 10	*n* = 11	
Operative time minutes	22 ± 11	21.6 ± 11	0.25
Time to discharge (hours)	7 ± 5	6 ± 5	0.16
Local anesthetic wound infiltration, *n* (%)	104 (100)%	103 (100)%	

Data are expressed as mean ± standard deviation when applicable.

**Table 2:** Outcomes and parental satisfaction.

	Nonnarcotic (*n* = 104)	Narcotic (*n* = 103)	*P* value
Number of medication days	1.9 ± 1	1.8 ± 1.2	0.95
Time to normal activity (days)	4.5 ± 3.2	5.0 ± 4	0.92
Parental satisfaction, *n* (%)	101 (97%)	93 (90%)	0.049

Data are expressed as mean ± standard deviation when applicable.
